# Treatment-Induced Changes in Plasma Adiponectin Do Not Reduce Urinary Albumin Excretion in the Diabetes Prevention Program Cohort

**DOI:** 10.1371/journal.pone.0136853

**Published:** 2015-08-27

**Authors:** Kieren J. Mather, Qing Pan, William C. Knowler, Tohru Funahashi, George A. Bray, Richard Arakaki, Bonita Falkner, Kumar Sharma, Barry J. Goldstein, DPP Research Group

**Affiliations:** 1 Division of Endocrinology and Metabolism, Indiana University, Indianapolis, Indiana, United States of America; 2 The Biostatistics Center, The George Washington University, Rockville, Maryland, United States of America; 3 Diabetes Epidemiology and Clinical Research Section, National Institute of Diabetes and Digestive and Kidney Diseases, Phoenix, Arizona, United States of America; 4 Department of Internal Medicine and Molecular Science, Graduate School of Medicine, Osaka University, Suita, Osaka, Japan; 5 Pennington Biomedical Center, Baton Rouge, Louisiana, United States of America; 6 Dept. of Medicine, Diabetes and Endocrinology, University of Hawaii, Honolulu, Hawaii, United States of America; 7 Department of Medicine, Thomas Jefferson University, Philadelphia, Pennsylvania, United States of America; 8 Division of Nephrology and Hypertension and Center for Renal Translational Medicine, Department of Medicine, University of California San Diego/Veterans Affairs San Diego Healthcare System, San Diego, California, United States of America; 9 Division of Endocrinology, Diabetes and Metabolic Diseases, Department of Medicine, Jefferson Medical College, Thomas Jefferson University, Philadelphia, Pennsylvania, United States of America; German Diabetes Center, Leibniz Center for Diabetes Research at Heinrich Heine University Duesseldorf, GERMANY

## Abstract

**Background and Objectives:**

Molecular data suggests that adiponectin may directly regulate urinary albumin excretion. In the Diabetes Prevention Program (DPP) we measured adiponectin and albuminuria before and after intervention, and we previously reported increases in adiponectin with interventions. Here we have used the DPP dataset to test the hypothesis that treatment-related increases in adiponectin may reduce albuminuria in obesity.

**Design, Setting, Participants and Methods:**

We evaluated cross-sectional correlations between plasma adiponectin and urinary albumin excretion at baseline, and the relationship of treatment-related changes in adiponectin and albuminuria. Baseline and follow-up urine albumin to creatinine ratios (ACR (albumin to creatinine ratio)) and plasma adiponectin concentration were available in 2553 subjects.

**Results:**

Adjusting for age, sex and race/ethnicity, we observed a statistically significant but weak inverse relationship between adiponectin and ACR at baseline (conditional Spearman’s rho = (-) 0.04, p = 0.04). Although DPP treatments significantly increased plasma adiponectin, there were no treatment effects on ACR and no differences in ACR across treatment groups. There was a weak direct (not inverse) association between change in adiponectin and change in albuminuria (adjusted Spearman’s rho = (+) 0.04, p = 0.03).

**Conclusions:**

In a large, well-characterized cohort of obese dysglycemic subjects we observed a weak inverse association between circulating adiponectin concentrations and urinary albumin excretion at baseline. Contrary to the hypothesized effect, treatment-related increases in plasma adiponectin were not associated with a reduction in ACR. The association of change in adiponectin with change in ACR should be assessed in populations with overt albuminuria before excluding a beneficial effect of increasing adiponectin to reduce ACR in obesity.

## Introduction

Adiponectin is an adipocyte-derived protein that circulates in high abundance. Paradoxically, adiponectin concentrations are reduced with increasing obesity. Adiponectin can serve as a marker of obesity-associated health risks [1–3]. Moreover, adiponectin appears to be functionally important in the vasculature [4, 5].

A direct role for adiponectin in the regulation of glomerular function has recently been suggested. The adiponectin deficient mouse has increased urinary albumin excretion along with other renal abnormalities [6], and in the glomerulus adiponectin deficiency induces segmental fusion of foot processes, increased podocyte permeability, and albuminuria [7]. Therefore reduced adiponectin concentrations (as commonly seen in obesity) may contribute to albuminuria, and conversely interventions that improve adiponectin levels may help reduce albuminuria.

The Diabetes Prevention Program (DPP) was a multicenter, randomized, placebo-controlled clinical trial of the effect of intensive lifestyle modification or metformin versus placebo in the prevention of diabetes onset among high risk subjects. The majority of this cohort had urine albumin to creatinine ratios (ACR) below 30 mg/g [8]. In this cohort we have previously shown a direct relationship between urine ACR and obesity (BMI (body mass index) in quartile 1 of ACR 33±6, BMI in quartile 4 35±7; Spearman’s correlation coefficient 0.09, p<0.01) [8]. Separately, we have also reported significant effects of DPP interventions to increase adiponectin concentrations, which helped reduce diabetes incidence [1]. Here we have evaluated cross-sectional associations of adiponectin and albuminuria at baseline in the DPP cohort, and tested the hypothesis that treatment-related increases in adiponectin may reduce albuminuria in obesity.

## Methods

The design, methodology and main outcomes of the DPP have been published previously [9]. In the DPP, subjects with elevated fasting glucose (5.3–6.9 mmol/L, or <6.9 mmol/L for American Indians), impaired glucose tolerance (glucose 7.8–11.0 mmol/L 2 hours after a 75 g oral glucose load), and elevated body mass index (≥22 kg/m^2^ for Asian Americans, ≥24 kg/m^2^ for others) were randomly assigned to one of three treatments: intensive lifestyle intervention, treatment with metformin, or treatment with placebo [9]. An adaptive randomization method stratified by study site was applied, to ensure balance of treatment assignments across centers [10]. A fourth intervention arm using troglitazone was prematurely terminated; subjects from that arm were not included in the present analyses. The majority of the cohort was obese (mean BMI 34±7), and the cohort included approximately 55% of European descent, 20% African American, 16% Hispanic American, 5% Native American, and 4% Asian American participants. All participants received standard advice for healthy diet and physical activity, including recommendations to limit total caloric intake and to undertake regular physical exercise. Use of thiazide diuretics or beta adrenergic antagonists was an exclusion criterion, but other classes of antihypertensive agents were permitted. New medical therapies initiated by non-study physicians following enrollment were not restricted. Institutional Review Board approval was obtained by each participating site (see S2 Appendix), and all participants included in this report provided written informed consent for the main study and for subsequent investigations, including subsequent use of blood and urine samples for secondary analyses. The study was performed in accordance with the Declaration of Helsinki.

All measurements were made on singly-thawed samples, using a study-wide central laboratory (Northwest Lipid Metabolism and Diabetes Research Labs, Seattle WA) for storage and analyses. Urine samples were measured continuously as they arrived from study centers, using a single automated methodology throughout the analysis period. Measurements were made on blood samples collected following an overnight fast. Blood samples for adiponectin measures were taken from long-term storage in 2006, and analyzed in batches with baseline and year 1 measurements from a given individual analyzed within a single measurement panel.

Total circulating adiponectin was measured in plasma using a latex-particle enhanced turbidimetric assay (Otsuka Pharmaceutical, Tokyo). Details of the performance of this assay have been previously published [11]; within-run and total coefficient of variation for this assay were 0.8–1.9% and 1.1–2.0%, respectively.

Urine albumin excretion (albuminuria) was assessed in DPP as a measure of the impact of glycemia on kidney function. Singlet spot urine samples were collected at baseline and end-of-study visits for measurement of albumin (Dade Behring BN II nephelometer) and creatinine (Hitachi 917 autoanalyzer). Median follow-up time for the cohort used in our analysis is 3.1 (25^th^ and 75^th^ percentiles 2.5 and 3.8 years respectively), depending on timing of study entry [9]. The albumin to creatinine ratio was calculated as the mathematical ratio of the urine concentrations of these analytes. Estimated glomerular filtration rate (eGFR) was calculated using serum creatinine concentrations and the CKD-EPI (Chronic Kidney Disease Epidemiology collaboration) equation [12, 13]. We applied the KDOQI 2012 guideline parameters for categorizing degree of albuminuria [14], distinguishing values ≥ 30 mg/g (moderately severe albuminuria) from the normal range values below this threshold.

ACR and adiponectin exhibited skewed distributions, and the urine albumin concentrations used in calculating ACR are truncated for values below the detection limit. Therefore we evaluated unadjusted Spearman correlations, and conditional Spearman correlation coefficients adjusting for demographic factors (age, race/ethnicity, and sex), which exhibit important associations with adiponectin and/or ACR [1, 8]. Comparisons of variables across treatment groups were performed with Chi-square test of independence for categorical variables, analysis of variance (ANOVA) for normal-distributed continuous variables or Kruskal-Wallace tests for skewed ACR and adiponectin variables, testing whether distributions in the three groups were different. For analyses by baseline adiponectin quartile, where rank ties at the quartile cut-points occurred, tied observations were included in the upper category. Comparisons of the baseline adiponectin distribution across different levels of the categorical variables were performed using the Kruskal-Wallace test, and the relationship between baseline adiponectin and continuous variables were assessed using Spearman’s rank correlation coefficients. Comparisons of correlation coefficients across treatment groups were performed by applying a Fisher’s z-transformation of the correlation coefficient values, constructing contrasts comparing each transformed value to the mean of the transformed values from the three groups, and then evaluating whether these contrasts equal zero simultaneously. Quantile regression was used to perform multivariable analyses of determinants of change in ACR. Analyses were performed using SAS 9.2 (SAS Institute, Cary NC).

## Results

The total DPP cohort at randomization included 3234 participants in the three-arm randomized study [9]. ACR results were available in 3187 subjects at baseline (1064 lifestyle group subjects, 1054 metformin group subjects, and 1069 placebo group subjects). Paired baseline and end-of-study ACR measurements were available for 963 lifestyle subjects, 927 metformin subjects, and 929 placebo subjects. Baseline adiponectin measurements were available for 3072 subjects (1028 lifestyle subjects, 1014 metformin subjects, and 1030 placebo subjects) and measurements from samples taken at one year of treatment intervention were available in 2801 subjects (927 lifestyle subjects, 932 metformin subjects, and 942 placebo subjects). A total of 2553 participants had complete paired data (871 lifestyle, 837 metformin and 845 placebo group individuals). The baseline characteristics (Table 1) of this majority subset of the overall cohort did not differ from those of the complete cohort (not shown). All analyses presented include only the subset with both sets of measurements.

**Table 1:** Characteristics of the DPP cohort with evaluable adiponectin and albuminuria data at baseline and follow-up. The change in adiponectin was calculated as Year 1 minus Baseline. The change in ACR was calculated as DPP-end minus baseline (see Methods). The p value is for comparisons of each variable across the three treatment groups.

	Lifestyle (n = 871)	Metformin (n = 837)	Placebo (n = 845)	p value
	Median [25^th^, 75^th^] orMean (SD)	Median [25^th^, 75^th^] orMean (SD)	Median [25^th^, 75^th^] orMean (SD)	
**Baseline**				
Sex (n, % of each group)				0.41
Male	293 (34%)	296 (35%)	273 (32%)	
Female	578 (66%)	541 (65%)	572 (68%)	
Race (n, % of each group)				0.32
Caucasian	485 (56%)	467 (56%)	465 (55%)	
African American	149 (17%)	165 (20%)	163 (19%)	
Hispanic	136 (16%)	133 (16%)	130 (15%0	
Asian American	51 (6%)	26 (3%)	37 (4%)	
Native American	50 (6%)	46 (5%)	50 (6%)	
Age (yrs)	51.7 (11.1)	51.8 (10.0)	51.1 (10.1)	0.18
BMI (kg/m^2^)	33.5 (6.5)	33.7 (6.4)	34.1 (6.7)	0.04
Waist (cm)	105 (14)	105 (14)	105 (14)	0.33
HOMA-IR (U)	6.8 (3.9)	7.1 (4.1)	7.1 (4.3)	0.15
2hr OGTT glucose (mg/dL)	164 (17)	165 (17)	165 (17)	0.51
Serum Creatinine (mg/dL)	0.8 (0.2)	0.8 (0.2)	0.8 (0.2)	0.45
eGFR (ml/min/1.73 m^2^)	97.1 (16.6)	97.2 (16.3)	97.9 (16.4)	0.27
Adiponectin (μg/mL)	7.50 [3.63, 8.12]	7.40 [3.61, 8.16]	7.20 [3.32, 7.83]	0.20
ACR (mg/g)	5.45 [14.65, 54.06]	5.43 [13.80, 35.02]	5.57 [12.92, 58.60]	0.69
**Post-Treatment Results**				
Adiponectin (μg/mL)	8.30 [3.94, 8.93]	7.40 [3.61, 8.16]	7.20 [3.32, 7.83]	<0.001
ACR (mg/g)	5.78 [14.79, 85.44]	5.61 [14.04, 38.73]	5.61 [14.97, 47.61]	0.72
**Change During DPP**				
Adiponectin (μg/mL)	0.81 [0.60, 1.72]	0.20 [0.10, 1.45]	0.10 [0.00, 1.24]	<.0001
ACR (mg/g)	0.14 [0.07, 97.57]	0.24 [0.07, 37.34]	2.05 [0.17, 46.43]	0.52

ACR, albumin to creatinine ratio; BMI, body mass index; eGFR, estimated glomerular filtration rate; HOMA-IR, homeostasis model assessment of insulin resistance; OGTT, oral glucose tolerance test.

### Cross-sectional relationships of adiponectin and albuminuria

The distributions of baseline adiponectin concentrations and ACR in this population have been separately published [1, 8]; for the subset analyzed the mean, median, 25^th^ and 75^th^ percentiles for adiponectin were 8.04, 7.4, 5.6 and 9.7 μg/mL and for ACR were 13.8, 5.5, 3.8 and 9.6 mg/g). ACR exhibited a strongly skewed distribution. 25% of participants had ACR above 10 mg/g (mildly increased albuminuria)[14]. Very few participants had ACR values above the 30 mg/g diagnostic threshold for moderately increased albuminuria and none of our participants had evidence of severely increased albuminuria. Analyses were not materially affected by excluding participants with values above 30 (data not shown); analyses presented include these participants. Urinary albumin concentrations were below the limit of detection in 13.5% (438/3234) of subjects.

Table 2 presents clinical data and ACR values according to quartiles of adiponectin concentrations. At baseline, the unadjusted Spearman’s correlation coefficient between adiponectin concentrations and ACR was (+)0.02 (p = 0.29; Table 2). Age, sex and race/ethnicity have known relationships with adiponectin concentrations and with ACR in this cohort [1, 8]; adjusting for these potentially confounding variables produced a conditional Spearman correlation coefficient of (-)0.04 (p = 0.04; Table 2).

**Table 2:** Relationships of albuminuria, renal function, and metabolic variables with adiponectin concentrations at baseline. All variables are presented as mean (SD) except as noted for ACR, which is highly skewed. Spearman’s correlations are presented for continuous variables versus baseline adiponectin levels. For categorical variables such as race and sex, the distributions of adiponectin within each category were compared using the Kruskal-Wallace test (denoted by *). Results are presented for unadjusted correlations, and following adjustment for race/ethnicity, sex and age. The numbers of individuals in each quartile are unequal due to ties, which are included in the higher quartile.

	Adiponectin Quartile					
	1	2	3	4	Unadjusted (p value)	Adjusted (p value)
N	625	650	625	653		
Adiponectin (μg/mL)	4.5 (0.8)	6.5 (0.5)	8.4 (0.7)	12.7 (3.3)	-	-
Sex					<0.001	
Male	321 (51%)	197 (38%)	172 (28%)	119 (18%)		
Female	304 (49%)	400 (62%)	453 (72%)	534 (82%)		-
Race (n, % of each quartile)					<0.001*	-
Caucasian	257 (41%)	359 (55%)	360 (58%)	441 (68%)		
African American	191 (31%)	115 (18%)	95 (15%)	76 (12%)		
Hispanic	100 (16%)	100 (15%)	103 (16%)	96 (15%)		
Asian American	43 (7%)	36 (6%)	28 (4%)	7 (1%)		
Native America	34 (5%)	40 (6%)	39 (6%)	33 (5%)		
Age	48.4 (9.7)	49.8 (10.2)	52.1 (10.3)	55.7 (9.9)	0.28 (<0.001)	-
BMI (kg/m^2^)	34 (6)	34 (7)	34 (6)	33 (6)	-0.07 (0.0003)	-0.08 (0.0002)
Waist (cm)	107 (14)	107 (15)	105 (14)	101 (13)	-0.16 (<0.0001)	-0.11 (<0.0001)
HOMA-IR (U)	9 (5)	7 (4)	7 (4)	5 (3)	-0.34 (<0.0001)	-0.31 (<0.0001)
2hr OGTT glucose (mg/dL)	165 (17)	165 (17)	165 (17)	164 (17)	-0.03 (0.19)	-0.05 (0.019)
eGFR (mL/min/1.73m^2^)	101 (17)	99 (16)	97 (16)	93(16)	-0.19 (<0.0001)	-0.02 (0.29)
Serum Creatinine (mg/dL)	0.82 (0.18)	0.79 (0.18)	0.77 (0.17)	0.76 (0.16)	-0.14 (<0.0001)	0.01 (0.52)
ACR (mg/g)	5.40 [3.55, 10.59]	5.39 [3.79, 9.47]	5.52 [3.79, 9.03]	5.69 [3.85, 9.83]	0.02 (0.29)	-0.04 (0.04)
(Median [25^th^, 75th])						

ACR, albumin to creatinine ratio; BMI, body mass index; eGFR, glomerular filtration rate estimated using the CKD-EPI equation; HOMA-IR, homeostasis model assessment index of insulin resistance; OGTT, oral glucose tolerance test.

The age/sex/race adjusted Spearman correlation coefficient between BMI and ACR was (+)0.09 (p<0.001). Further adjusting for the contribution of adiponectin did not materially change this relationship (r = (+)0.09, p<0.001). Similarly, adjusting the relationship of adiponectin with ACR for the effect of BMI also did not materially change the relationship (r = (-)0.03, p = 0.08). Obesity and circulating adiponectin concentrations therefore affect ACR independent of one another in this cohort.

In view of a prior report of an inverse association between adiponectin concentration and ACR specifically in an African American cohort [7], we undertook post-hoc analyses evaluating these relationships within race/ethnicity subgroups. These subgroups did not differ in their relationship between adiponectin and albuminuria (p = 0.48 comparing groups in unadjusted analyses). Within each race/ethnicity subgroup the overall result was concordant with the whole-cohort finding, reaching significance only among white participants (the largest single subgroup, 1417 participants; adjusted r value (-)0.06, p = 0.02). The adjusted correlation coefficient among African American participants (477 participants) was (-)0.015, p = 0.74.

Women comprised about 2/3 of our study population and the overall study result was seen among the women in our study (adjusted Spearman r value (-)0.06, p = 0.009). In the adjusted analyses there was no significant sex difference in the relationship of adiponectin with ACR (p = 0.09 comparing sexes).

These associations were evaluated within age subgroups (<45, 45–59, ≥60 years), with no significant associations evident within each age subgroup and no difference in the relationships when comparing these groups (p = 0.77).

Baseline associations were present between blood pressure, ACR and adiponectin concentrations (Adiponectin versus DBP (diastolic blood pressure) r = (-)0.09, p<0.001; ACR versus SBP (systolic blood pressure) r = (+)0.21, p<0.001; ACR versus DBP r = (+)0.12, p<0.001). Adding adjustment for DBP to the age/sex/race adjusted model did not materially change the apparent relationship between adiponectin and ACR (Spearman’s r = (-)0.04, p = 0.04).

At baseline, self-reported use of angiotensin converting enzyme inhibitors (ACEI) or angiotensin receptor blockers (ARB) was associated with ACR (r = (+)0.06, p = 0.004). However, there was no relationship of ACEI or ARB use with adiponectin concentrations. Therefore we did not adjust the above analyses for use of these medications

### Effects of Intervention-related changes in adiponectin on albuminuria

At study baseline, there were no differences across treatment groups in ACR or prevalence of moderately severe albuminuria (previously denoted ‘microalbuminuria’) [14], with a study-wide prevalence of 6.4%. At the end of the study, the prevalence of moderately severe albuminuria (ACR ≥ 30 mg/g) was 7.0%, 6.5%, and 5.6% in placebo, metformin, and lifestyle groups respectively (p = 0.51 comparing treatment groups). Median ACR values did not differ across groups at end of study (5.8, 5.6, and 5.6 mg/g for placebo, metformin, and lifestyle, p = 0.72). There was no significant change in ACR with intervention within the DPP, and change in ACR was not significantly different across treatment groups (Table 1).

The magnitudes of change in adiponectin and ACR are reported in Table 1; the change in adiponectin differed across treatment groups but the change in ACR did not (as we have previously reported for each finding individually [1, 15]). The central hypothesis for the current analyses is that treatment-related increases in adiponectin may reduce albuminuria in obesity. We therefore evaluated whether the change in adiponectin was associated with change in ACR (Table 3). All correlation coefficients were positive, indicating concordant (not inverse) changes in ACR and adiponectin; these correlations were not statistically significant within treatment groups, but did achieve significance when all treatment groups were pooled. The magnitude of this association was small. The the coefficients and significance were unaffected by adjustments for age, race, and sex. In exploratory quartile regression analyses, we found that lower baseline ACR, greater change in DBP and female sex were significantly associated with increase in ACR; specifically, baseline adiponectin, change in adiponectin, baseline BMI, race/ethnicity and baseline use of angiotensin converting enzyme inhibitor were not significantly related to change in ACR in these analyses.

**Table 3:** Correlations of Changes in Adiponectin and Changes in Albumin/creatinine ration (ACR). Adiponectin concentrations were measured at baseline and at Year 1 of intervention. ACR was measured at baseline and at end-of-study. Values presented are Spearman’s correlation coefficient ρ (p value), except the column testing group differences in the correlation. Adjustments include age, race and sex.

	Lifestyleρ (p value)N = 871	Metforminρ (p value)N = 837	Placeboρ (p value)N = 845	p value comparing groups	Pooled correlationρ (p value)N = 2553
Unadjusted	0.06 (0.09)	0.05 (0.16)	0.02 (0.50)	**0.77**	0.04 (0.03)
Adjusted	0.06 (0.09)	0.05 (0.14)	0.02 (0.56)	**0.71**	0.04 (0.03)

## Discussion

Recent reports of direct functional effects of adiponectin on podocyte function suggest that interventions that augment adiponectin concentrations might help reduce albuminuria. In this context, we have evaluated the relationships between circulating adiponectin concentration and urinary albumin to creatinine ratio (ACR) at baseline, and the changes in these variables with study interventions, among more than 2500 obese normo-albuminuric participants in the Diabetes Prevention Program. In this large, well validated cohort we found a statistically significant but weak inverse relationship between adiponectin and ACR at baseline. Despite beneficial effects of DPP treatments to increase adiponectin, there was no evidence for a treatment effect on albuminuria and no evidence for an inverse relationship between treatment-induced change in adiponectin and change in albuminuria. To the contrary, we observed a statistically significant direct correlation of change in adiponectin with change in ACR, arguing strongly against an effect of increased adiponectin to reduce albuminuria in this population.

### Cross-Sectional Relationships

The epidemiologic association between circulating adiponectin concentrations and measures of urinary protein excretion differs according to different stages of renal disease [6, 16–21]. Within low-albuminuric populations with dysglycemia the data are inconsistent. Most groups have observed an inverse relationship as we have seen here [7, 22–26], but the literature also includes reports of absent or even direct relationships [18, 27, 28]. A recent publication evaluated associations of molecular weight isoforms of adiponectin with albuminuria in a normo-albuminuric non-obese European white population, and found direct (not inverse) relationships of high molecular weight adiponectin with ACR, although with a very modest slope [28]. In that report, other isoforms were not significantly associated with ACR. The FinnDiane Study Group [18] describes comparable adiponectin concentrations in the moderately severe (micro)-albuminuric group (12.1±5.8 mg/dL) versus the normo-albuminuric group (12.3±6.2), both lower than the severely albuminuric group (18.1±12.0). Similar patterns are evident in other reports in subjects with diabetes [16, 17]. Although the coefficients for these associations were not formally reported for each subgroup, there do not appear to be differences in circulating adiponectin concentrations between normo-albuminuric and moderately albuminuric subject groups in these reports. In a cohort of 440 obese people of European descent, 80% women, the correlation between adiponectin and ACR was (-)0.26, concordant with our current observations. By virtue of the design of the DPP, we have data from a large, well-defined obese dysglycemic (but non-diabetic) population. Our observation of a weakly inverse but statistically significant cross-sectional relationship is consistent with prior observations of inverse relationships, and our large sample size provides confidence in the precision of the estimate. However, this association is weak and therefore the relationship between low adiponectin and urinary albumin excretion is of questionable physiologic and epidemiologic relevance, at least in obese populations with normo-albuminuria.

### Treatment-Induced Changes

The assessment of inter-relationships of change in these variables with DPP interventions is more informative in terms of evaluating physiologic interactions between adiponectin and albuminuria. There is good reason to expect that change in adiponectin will directly affect albuminuria: Whole-animal genetic deficiency of adiponectin is associated with albuminuria and multiple renal abnormalities [6]; adiponectin was recently shown to be directly involved in podocyte structure and function in an in vitro model [7]; and adiponectin overexpression/underexpression directly affects renal response to a genetically induced renal injury [29]. Also, low baseline adiponectin concentrations were predictive of progression of albuminuria in a T2D cohort with elevated baseline albuminuria [30]. In the DPP, all 3 treatment interventions (placebo, metformin, lifestyle) were associated with increases in circulating adiponectin and these changes were proportional to weight loss and to the diabetes prevention effects of the interventions (1). Despite these treatment-induced changes in adiponectin concentration, we found no differences in albumin excretion by treatment group, and we found no evidence for an inverse relationship between change in adiponectin and change in albuminuria. In fact, we observed a statistically significant direct (not inverse) correlation between changes in these parameters.

Surgically-induced weight loss can reduce urinary albumin excretion in obese diabetic subjects [31]; it is possible that this effect of weight loss is mediated by adiponectin. However, the DPP interventions did not produce any measurable reduction in albuminuria despite significant weight loss and the attendant increases in circulating adiponectin. The antidiabetic agents rosiglitazone (a PPARgamma agonist) and miglitol (an alpha-glucosidase inhibitor) have been found to concordantly improve adiponectin and urinary albumin excretion [32, 33], and it is possible that these observations reflect beneficial actions of adiponectin on albuminuria. The current observations suggest that treatment-induced changes in adiponectin cannot be expected to reduce albuminuria within the normo-albuminuric range. Further studies specifically evaluating treatment-induced changes in adiponectin with treatment effects on albumin excretion in populations with at least moderately severe albuminuria will be needed to understand whether the relationships predicted from molecular studies are important in states with more overtly abnormal albuminuria.

### Strengths and Limitations

Two main strengths of our study are noteworthy. First, we studied the relationships of adiponectin and albuminuria in the largest cohort of normo-albuminuric subjects reported to date. Second, our cohort is multi-ethnic and therefore broadly representative.

Limitations also apply. Urine ACR values were measured in single, random spot urines; future studies with repeated timed urines may provide additional insights. Nevertheless, our observations are consistent with recent reports of inverse associations in non-obese normo-albuminuric populations [26, 28]. Since our study population was normo-albuminuric, failure to improve albuminuria from this already low range is perhaps not surprising; observations in this type of population have not been previously reported. The current findings may not reflect the relationship among populations with greater degrees of albuminuria.

### Summary

In a large, well-characterized cohort of obese dysglycemic subjects at risk for diabetes we found evidence for a statistically significant but weak inverse association between circulating adiponectin concentrations and urinary albumin excretion, measured as the albumin to creatinine ratio (ACR). DPP treatment interventions produced significant increases in circulating adiponectin, but these treatments were not associated with a significant change in ACR, and there was no evidence for treatment-induced increases in adiponectin to drive reductions in albuminuria. Further studies evaluating the effects of treatment-induced improvements in adiponectin and albuminuria are needed, in populations with manifest albuminuria.

## Supporting Information

S1 Appendix Diabetes Prevention Program Research Group list. (DOCX)

S2 Appendix List of supervising IRBs. (DOCX)
