# Supplementary material for: Treatment-Induced Changes in Plasma Adiponectin Do Not Reduce Urinary Albumin Excretion in the Diabetes Prevention Program Cohort
Source: PLoS One. 2015 Aug 27;10(8):e0136853. doi: 10.1371/journal.pone.0136853 (PMC4551844; doi:10.1371/journal.pone.0136853)
Supplement: S2 Appendix — (DOCX) [file pone.0136853.s002.docx]

**Appendix 2. List of supervising IRBs**

Study procedures and documents were approved by the following institutional review boards: Pennington Biomedical Research Center; University of Chicago; Kaiser Permanente, Thomas Jefferson University; University of Miami; University of Texas Healthcare System, San Antonio; Department of Veterans Affairs, San Antonio; Colorado Multiple; Joslin Diabetes Center Committee on Human Studies; Veterans Affairs Puget Sound Health Care System; University of Washington; University of Tennessee Health Science Center; Northwestern University; Partners Human Research Committee; University of California, San Diego; St. Luke's Roosevelt Institute for Health Sciences; Indiana University; Medstar Health Research Institute, Baltimore; Medstar Health Research Institute, Washington; University of California, Los Angeles; Washington University School of Medicine; Johns Hopkins Medicine; University of New Mexico; Albert Einstein College of Medicine; University of Pittsburgh; University of Hawaii; National Institute for Diabetes and Digestive and Kidney Diseases; and the Navajo Nation Human Research Review Board.
